# 90. Risk Factors for Community Colonization with Extended-Spectrum Cephalosporin-Resistant Enterobacterales (ESCrE) in Botswana An Antibiotic Resistance in Communities and Hospitals (ARCH) Study

**DOI:** 10.1093/ofid/ofac492.015

**Published:** 2022-12-15

**Authors:** Naledi Mannathoko, Mosepele Mosepele, Robert Gross, Rachel Mann Smith, Kevin Alby, Laurel Glaser, Melissa Richard-Greenblatt, Aditya Sharma, Anne Jaskowiak-Barr, Kgotlaetsile Sewawa, Laura Cowden, Emily Reesey, Dimpho Otukile, Leigh Cressman, Giacomo Paganotti, Margaret Mokomane, Ebbing Lautenbach

**Affiliations:** University of Botswana, Gaborone, Southern, Botswana; University of Botswana, Gaborone, Southern, Botswana; University of Pennsylvania, PHiladelphia, Pennsylvania; CDC, Atlanta, Georgia; University of North Carolina, Chapel Hill, North Carolina; University of Pennsylvania, PHiladelphia, Pennsylvania; University of Pennslvania, Philadelphia, Pennsylvania; CDC, Atlanta, Georgia; University of Pennslvania, Philadelphia, Pennsylvania; University of Botswana, Gaborone, Southern, Botswana; University of Pennsylvania, PHiladelphia, Pennsylvania; University of Pennsylvania, PHiladelphia, Pennsylvania; University of Botswana, Gaborone, Southern, Botswana; University of Pennsylvania, PHiladelphia, Pennsylvania; University of Botswana, Gaborone, Southern, Botswana; University of Botswana, Gaborone, Southern, Botswana; University of Pennsylvania, PHiladelphia, Pennsylvania

## Abstract

**Background:**

The epidemiology of ESCrE in low- and middle-income countries (LMICs) is poorly described. While risk factors for ESCrE clinical infection have been studied, little is known of the epidemiology of ESCrE colonization. Identifying risk factors for ESCrE colonization specifically is nonetheless critical to inform antibiotic resistance reduction strategies.

**Methods:**

This study was conducted in 6 clinics located in 3 districts in Botswana. In each clinic, we surveyed a random sample of outpatients. We also invited each enrolled clinic subject to refer up to 3 adults. Each adult clinic or community subject was also invited to refer their children. All subjects had rectal swabs collected which were inoculated onto chromogenic media for preliminary identification of ESCrE. Final identification and susceptibility testing were performed using MALDI-TOF MS and VITEK-2, respectively. Data were collected on demographics, comorbidities, antibiotic use, healthcare exposures, travel, and farm and animal contact. Subjects with ESCrE colonization (cases) were compared to non-colonized subjects (controls). Bivariable and multivariable analyses were conducted to identify risk factors for ESCrE colonization.

**Results:**

Enrollment occurred from 1/15/20–9/4/20 and 2,000 subjects were enrolled. There were 959 (48.0%) clinic subjects, 477 (23.9%) adult community subjects, and 564 (28.2%) child community subjects. 725 (36.3%) subjects lived in the same household as another subject. The median (IQR) age was 30 (12–41) and 1,463 (73%) were female. There were 555 cases and 1,445 controls (i.e., 27.8% of subjects were ESCrE colonized). Unadjusted comparisons are noted in Table 1. Independent risk factors for ESCrE were younger age, hospital exposure, travel, and presence of an ESCrE colonized household member (Table 2).

**Conclusion:**

ESCrE colonization was common and associated with several exposures. Our results suggest even modest healthcare exposure may be important in driving ESCrE. The strong link to household member ESCrE colonization highlights the potential role of household transmission or common exposure. These findings warrant further prospective studies and provide vital information to inform strategies to curb further emergence of ESCrE in LMICs.

**Disclosures:**

**All Authors**: No reported disclosures.

